# Electro-active Variable-Stiffness Corrugated Structure Based on Shape-Memory Polymer Composite

**DOI:** 10.3390/polym12020387

**Published:** 2020-02-08

**Authors:** Xiaobo Gong, Fang Xie, Liwu Liu, Yanju Liu, Jinsong Leng

**Affiliations:** 1 School of Naval Architecture and Ocean Engineering, Harbin Institute of Technology at Weihai, Weihai 264209, China; fangxie@hit.edu.cn; 2 School of Astronautics, Harbin Institute of Technology, Harbin 150001, China; liuliwu_006@163.com (L.L.); yj_liu@hit.edu.cn (Y.L.); 3 Science and Technology on Advanced Composites in Special Environments Key Laboratory, Harbin Institute of Technology, Harbin 150001, China; lengjs@hit.edu.cn

**Keywords:** shape-memory polymer, shape-memory polymer composite, corrugated structure, morphing skin, SMP

## Abstract

Shape-memory polymers (SMPs) can adjust their stiffness, lock a temporary shape, and recover the permanent shape upon an appropriate stimulus. They are applied in the field of morphing skins. This work presents a variable-stiffness corrugated sheet based on a carbon fiber felt (CFF)-reinforced epoxy-based SMP composite that shows variable stiffness and extreme mechanical anisotropy for potential morphing skin applications. The corrugated sheet exhibits a variable stiffness with a change in temperature, which can help the skin adjust its stiffness according to different service environments. The corrugated sheet can be electrically heated rapidly and homogeneously due to its high electrical conductivity and enhanced heat transfer efficiency. Its Joule-heating effect acts as an effective active stimulation of the variable stiffness and shape-memory effect. The CFF-reinforced epoxy-based SMP composite was manufactured into a corrugated shape to obtain extreme mechanical anisotropy. The corrugated sheet shows a low in-plane stiffness to minimize the actuation energy, while it also possesses high out-of-plane stiffness to transfer the aerodynamic pressure load. Its mechanical properties, electrical heating performance, and shape-memory effect were investigated using experiments. The results show that the proposed SMP composite exhibits extreme mechanical anisotropy, considerable deformation ability, and variable stiffness induced by Joule heating without an external heater.

## 1. Introduction

Morphing aircraft can adapt their aerodynamic and structural shape to extend the aeromechanic flight envelope and accomplish multi-mission profiles; this attribute is treated as an important direction for future aircraft development [1,2,3,4,5]. Ingenious morphing wing structures are critical to realizing the characteristics of a morphing aircraft. The morphing wing structures are complex and sophisticated systems, including morphing skins, actuators, adaptive structures, and associated mechanisms. Each component should be designed for a performance trade-off between compliance to perform the morphed shape, stiffness to withstand and transfer the aerodynamic loads, and weight to maximize payloads while minimizing the airframe weight [2]. Therefore, a morphing skin should feature not only flexible properties to obtain large deformations and minimize driving forces, but also possess high stiffness to withstand and transfer aerodynamic pressure loads.

Variable-stiffness materials and structures aroused the interest of researchers to solve the contradiction between high stiffness and reversible deformability required by morphing skins [6]. Shape-memory polymers (SMPs) perform considerable stiffness variations under appropriate external stimulus, and they were chosen and investigated as morphing skins [7,8,9,10]. SMPs generally have typical transition temperatures (*T_trans_*). They are stiff below *T_trans_* and become flexible and possess high deformation capability when heated above *T_trans_*. In chemically cross-linked SMPs, *T_trans_* originates from *T_m_* (if the reversible phase is a semi-crystalline segment) or *T_g_* (if the SMP is a conventional thermoset) [11]. The epoxy-based SMP (written as SMPE in this paper) referred to in this work is an amorphous thermoset, which has a glass transition temperature (*T_g_*) for its shape-memory behavior.

Thermal stimulation is the most frequently used activation method for SMPs [12]. Electrical Joule resistive heating of the conductive composite can be a desirable stimulus to activate SMPs without external heating equipment. Several conductive SMP composites consisting of conductive fillers were fabricated and investigated [13,14], such as carbon nanotubes [15,16], graphene [17,18,19], carbon nanoparticles [20,21,22], ferromagnetic particles [23], carbon nanopaper [24], and continuous carbon fiber [25]. Due to high raw material and manufacturing costs and low stability, the generally used actuation methods in engineering applications are external heating devices, for example, nickel–chromium alloy heating wires and heating films integrated with heating wires and polyimide films [26,27]. When a heating wire is used for heating, the heat distribution is not uniform. The temperature near the heating wire is high, and the temperature away from the heating wire is almost unchanged. Furthermore, because the diameter of the heating wire is almost equal to the thickness of the SMP skin, the SMP would debond from the heating wires and crack after repeated heating and deformation. When using a heating film integrated with heating wires and polyimide films, the heating uniformity is improved, but the deformation ability is significantly limited. The problem of uniform and effective heating becomes the main factor limiting the application of SMP morphing skins. Carbon fiber felt (CFF)-reinforced SMPE composites can be rapidly and homogeneously electrically heated due to their high electrical conductivity and enhanced heat transfer efficiency [28].

In addition to the excellent electrical heating performance, CFF/SMPE plates have a limited elongation capacity and low loading capacity, especially when above *T_g_* [28]. The corrugated structure is a typical structure with extreme anisotropy, i.e., lateral flexibility and longitudinal rigidity. Corrugated structures are widely used in the packaging, civil engineering, and aerospace industry [29]. Its morphing skin application was firstly proposed by Yokozeki et al. [30]. The mechanical properties of corrugated sheets were investigated through theoretical and experimental analysis [31]. Several corrugated structures with various geometries and materials were later designed and proposed as morphing skins [32,33,34,35,36,37]. Corrugated structures were mainly proposed for morphing skins in chordwise [37,38,39], spanwise [40], and winglet morphing [41]. The overall results show that corrugated structures represent a potential morphing skin at low speeds for small air vehicles [29,42]. The CFF/SMPE composite can be easily processed into various structural forms, benefitting from its simple manufacturing process. A corrugated structure possesses not only elastic properties to obtain large deformations, but it also features high stiffness to withstand and transfer aerodynamic pressure loads.

This work firstly presents the fabrication and experimental analysis of variable-stiffness corrugated sheets made of CFF/SMPE composite for morphing skin applications. The corrugated sheets exhibit both excellent variable stiffness properties and extreme mechanical anisotropy. Their Joule-heating effect acts as an effective active stimulation for the variable stiffness and shape-memory effect, benefitting from the electrical conductivity. The mechanical properties, electrical heating performance, and shape-memory effect were investigated using experiments.

## 2. Experimental Section

### 2.1. Fabrication of Corrugated Sheets

The variable-stiffness corrugated sheets were made of CFF/SMPE composite. The introduction of CFF greatly reduces the resistance of the SMPE composite, such that a corrugated sheet can be triggered by its own resistance under a certain voltage. The SMPE consists of epoxy resin, curing agent, and a linear monomer [43]. The linear monomer has a long molecular chain, and the main chain is mainly composed of carbon–oxygen bonds. The epoxy resin and linear monomer contain epoxy functional groups in the same molar ratio, and the molecular weight ratio of the two is 2:5. The ratio of epoxy resin and curing agent is 1:1, and the linear epoxy monomer accounts for 5 wt.% of the SMPE system. The CFF was purchased from Kaifeng Pengyuan Glass Fiber Products Co., Ltd. (Kaifeng, China) It is a non-woven fabric composed of randomly oriented carbon fiber, with a 30 g/m^2^ areal density and less than 5% organic content by weight. The CFF has a 7.02-μm average fiber diameter, and a 207-GPa elongation modulus.

**Figure 1:**
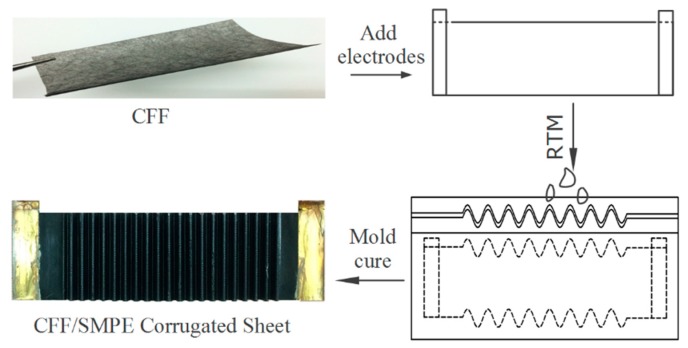
A schematic of the variable-stiffness corrugated sheet preparation.

The variable-stiffness corrugated sheet was made using a resin transfer molding (RTM) process with a set of corrugated shaped molds. The preparation schematic is shown in Figure 1. The corrugated mold set consisted of male and female molds with a corrugated mating surface with a sinusoidal shape and a 10-mm wavelength L, 3-mm amplitude H/2, 20 sinusoidal periods, and 75-mm width. Firstly, the CFF was cut into a 60 × 400 mm^2^ rectangle, and then copper foil was adhered to the CFF as an electrode using a conductive silver paste (HS-100RF, Kunshan Hisense Electronics Co., Ltd., Kunshan, China) with a 20-mm overlap width. The CFF with electrodes was heated to 135 °C for 1 h to completely cure the conductive paste for a better connection between CFF and the copper foil. The CFF sheet was pre-soaked with SMPE resin at room temperature for 2 h to fully infiltrate the felt. Subsequently, the infiltrated CFF was laid in the corrugated mold, and the epoxy-based SMPE resin was injected into the cavity. The SMPE composite sample was cured in a drying oven at 80 °C for 3 h, 100 °C for 3 h, and 150 °C for 5 h. After the curing process, a 0.36-mm-thick electrically driven corrugated sheet was obtained, as shown in Figure 1.

### 2.2. Characterization Methods

#### 2.2.1. Dynamic Mechanical Properties

The dynamic mechanical properties of SMPE and CFF/SMPE composite were studied using a dynamic mechanical analyzer (DMA, Mettler-Toledo AG Analytical, Zurich, Switzerland). The tests were performed in three-point bending mode using rectangular strip specimens with 9 × 3 × 1 mm^3^ dimensions. Three test pieces were selected for each test to ensure test repeatability. The storage modulus and tangent delta at different temperatures were obtained under a dynamic thermal scan. The heating rate was 5 °C/min from 40 to 180 °C, and the oscillation frequency was 1 Hz. The tangent delta is defined as the ratio of the loss modulus to the storage modulus. In this study, the peak of the tangent delta versus temperature curve was used to define *T_g_*.

#### 2.2.2. Quasi-Static Mechanical Properties

The mechanical property test was performed on a Zwick/Roell servo test machine (Zwick/Roell, Ulm, Germany), and the tensile rate and the three-point bending head drop rate were 3 mm/min and 1 mm/min, respectively. To verify the variable stiffness performance of the corrugated sheets, two ambient temperatures were selected for testing, 20 and 100 °C (*T_g_* of SMPE). Three test pieces were selected for each test to ensure test repeatability. The tensile test specimens had a length of 20 corrugation cycles of 160 mm and a width of 27.4 mm. The three-point bending test specimens had a lateral length of 24 mm and a longitudinal width of 60 mm. The span of the three-point bending test was 48 mm.

#### 2.2.3. Temperature Distribution

The temperature distribution on the surface of the corrugated sheet was recorded using an infrared camera (VarioCAM^®^ HiRessl, JENOPTIK Infra Tec., Dresden, Germany). The thermal images were recorded every second. The average temperature of the corrugated sheet was calculated using the software provided with the camera.

#### 2.2.4. Shape-Memory Effect

**Figure 2:**
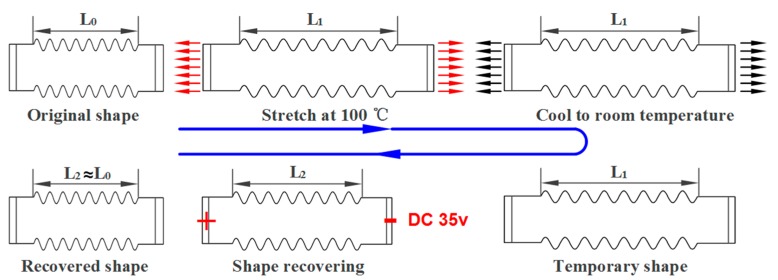
Schematic of the shape memory effect test of the carbon fiber felt-reinforced epoxy-based shape-memory polymer (CFF/SMPE) corrugated sheet.

The tensile shape recovery test using rectangular corrugated sheet specimens was used to verify the electro-activated shape-memory effect (SME) of the corrugated sheet. The schematic of the test is shown in Figure 2. The specimen had an initial length of L_0_ and was firstly stretched to L_1_, the temporary shape, at 100 °C (*T_g_*). The stretched temporary shape was locked by cooling it to room temperature. A 35-V constant direct current (DC) voltage was applied to the two electrodes of the stretched specimen to trigger the shape recovery. The length of the recovered specimen was denoted as L_2_, and the shape recovery ratio was calculated as R_r_ = (L_1_ − L_2_)/(L_1_ − L_0_) [12,44,45]. The infrared temperature camera recorded the temperature distribution during the shape recovery process.

## 3. Results and Discussion

### 3.1. Thermal Mechanical Properties

**Figure 3:**
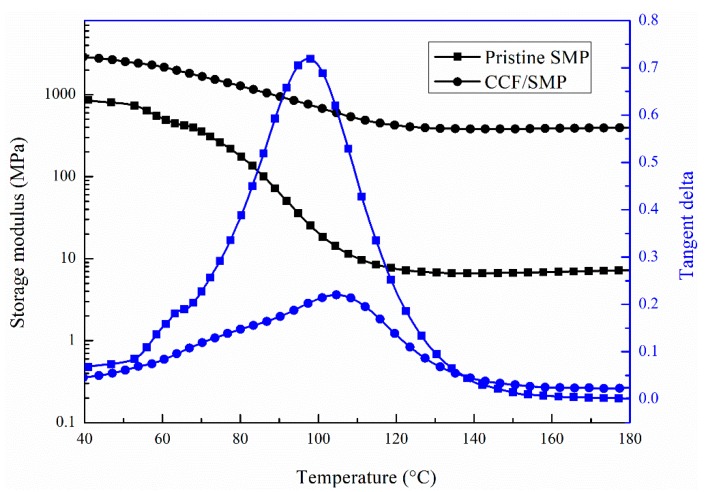
Curves of storage modulus and tangent delta versus temperature of pure SMPE and CFF/SMPE composite.

The storage modulus and tangent delta of SMPE and CFF/SMPE composites at different temperatures are shown in Figure 3. A decreasing trend in the storage modulus within the whole temperature range can be observed in this figure. The storage modulus dropped significantly in the region between 80 and 110 °C, which is believed to correspond to the glass transition region of SMPE. The higher values below 80 °C and sharp decrease in the values between 80 and 110 °C were due to the fact that the material was in a glassy state below 80 °C, during which the contribution of the elastic modulus was more than the viscous modulus, whereas the material was in glass transition stage between 80 and 110 °C, during which a change from glass state into rubber state took place. The storage modulus curves were almost straight lines with a slight decrease between 110 and 180 °C, which shows a rubbery regime, indicating degradation of the moduli above 110 °C. When the time scale of molecular motion coincided with that of mechanical deformation, each oscillation was converted into the maximum possible internal friction and nonelastic deformation [46]. There was a notable increase in the modulus of CFF/SMPE composite with the incorporation of CFF, due to the reinforcing effect imparted by the carbon fibers, allowing a greater degree of stress transfer at the interface [47].

The tangent delta is defined as a loss factor, i.e., the ratio of loss modulus to storage modulus, which shows the behavior of the damping factor for CFF and CFF/SMPE composites with an increase in the temperature. The damping properties of the material give the balance between the elastic phase and the viscous phase in a polymeric structure [48]. A higher value of tangent delta indicates that the material is in the viscous phase, whereas a lower value indicates that the material is in the elastic phase. The peak value of the tangent delta of CFF/SMPE composite decreased significantly with CFF incorporation. A low loss factor means a low internal energy loss and low damping property in dynamic deformation. The reduction for the CFF/SMPE composite was probably due to the strengthening effect of carbon fibers, limiting the mobility of the SMPE matrix [49]. The carbon fibers contained in CFF acted as barriers to the mobility of the polymer chain, leading to a lower degree of molecular motion and, hence, lower damping characteristics [50]. Another possible reason is that there was less matrix by volume to dissipate the vibration energy [49]. In this study, the peak value of the tangent delta is defined as *T_g_*. The peak values of the tangent delta of pure SMPE and CFF/SMPE composite occurred at 97.3 ± 3.8 °C and 104.7 ± 4.3 °C, respectively. They were both around 100 °C, which indicates that the addition of CFF had little influence on the *T_g_* of CFF/SMPE composite.

### 3.2. Quasi-Static Mechanical Properties

**Figure 4:**
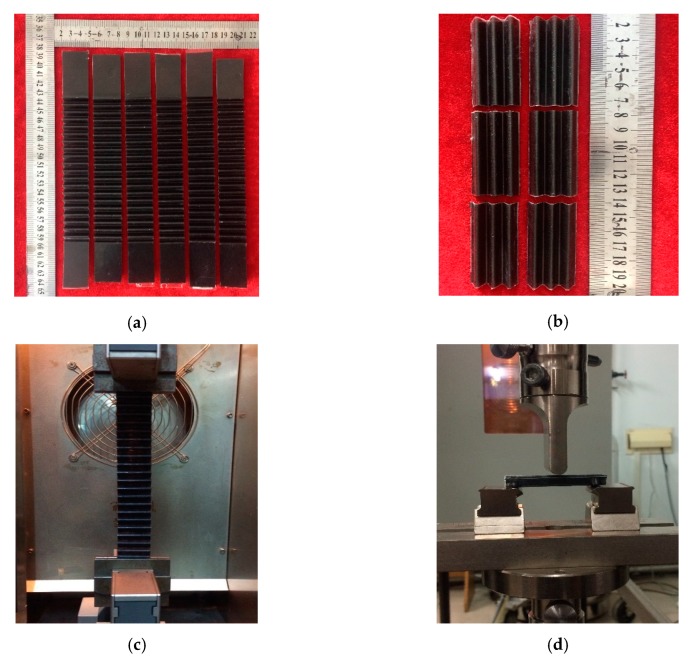
Specimens and experimental set-up for tensile test and three-point bending: (**a**) specimens for tensile test; (**b**) specimens for three-point bending test; (**c**) tensile test; (**d**) three-point bending test.

The SMPE composite has much higher Young’s modulus, tensile strength, and lower breaking strain than pure SMPE due to the introduction of CFF, based on the mechanical property test results in the previous work [28]. The transverse tensile stiffness and longitudinal bending stiffness are the main parameters that should be considered when using as morphing skin. The transverse tensile stiffness is closely related to its lateral deformation capacity and driving force requirement. The longitudinal bending stiffness determines the bearing capacity of the skin. In this study, the transverse tensile stiffness and longitudinal bending stiffness of the corrugated sheets were investigated with the tensile test and three-point bending test. The tensile test specimens and the three-point bending test specimens are shown in Figure 4a,b, respectively. The tensile test and the three-point bending test performed on a Zwick/Roell servo test machine are shown in Figure 4c,d, respectively.

**Figure 5:**
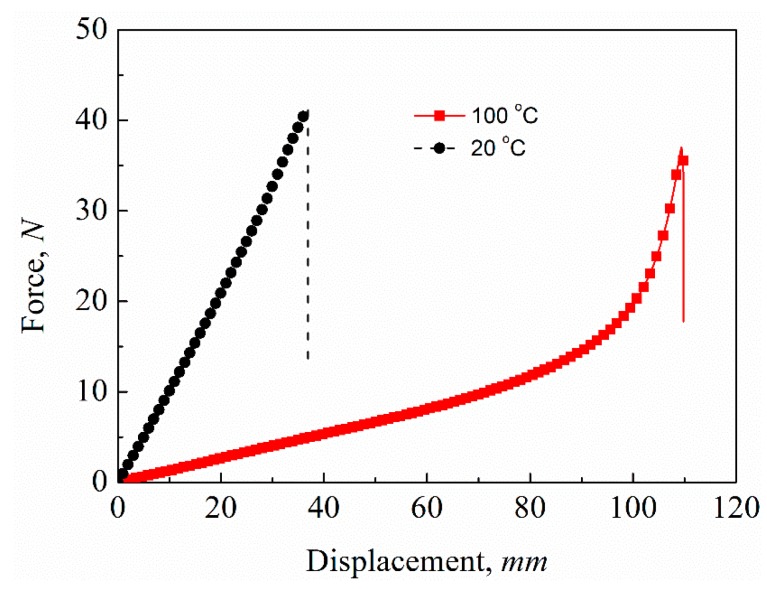
Tensile force–displacement curves of variable-stiffness corrugated sheets.

The force–displacement curves of the transverse tensile tests of the variable-stiffness corrugated sheets at different temperatures are shown in Figure 5. The test curves of the three test pieces are almost overlapping and show good repeatability under the same test conditions. Comparing the results at two temperatures, the transverse stiffness at room temperature, 20 °C, was higher, while the deformation capacity was smaller than at 100 °C. The average experimental data for tensile stiffness, force at break, and displacement at break at different temperatures are listed in Table 1, and the standard deviation of each configuration follows the ± sign. The force–displacement relationship was linear at room temperature, and the transverse tensile stiffness was 1.04 ± 0.06 N/mm. When the temperature reached the *T_g_* of SMPE (100 °C), the transverse tensile stiffness reduced drastically, while the deformation ability enhanced significantly. The force–displacement relationship was linear under small deformation, and the tensile stiffness was 0.15 ± 0.03 N/mm in this linear range. The transverse tensile stiffness at room temperature was 6.9 times that of *T_g_*, which shows the good variable stiffness property of the SMPE composite corrugated sheet. As the deformation increased, the curves changed non-linearly, and the transverse stiffness increased. When the corrugated sheet was stretched to a certain extent, the geometric shape of the corrugation changed substantially and the corrugated structure tended to be flattened [29], which produced an increase in tensile stiffness. Compared with the CFF-reinforced SMPE, the corrugated sheet had a much better deformation ability. The fracture strain was increased from less than 3% [28] to 23% at 20 °C and 68% at 100 °C.

**Figure 6:**
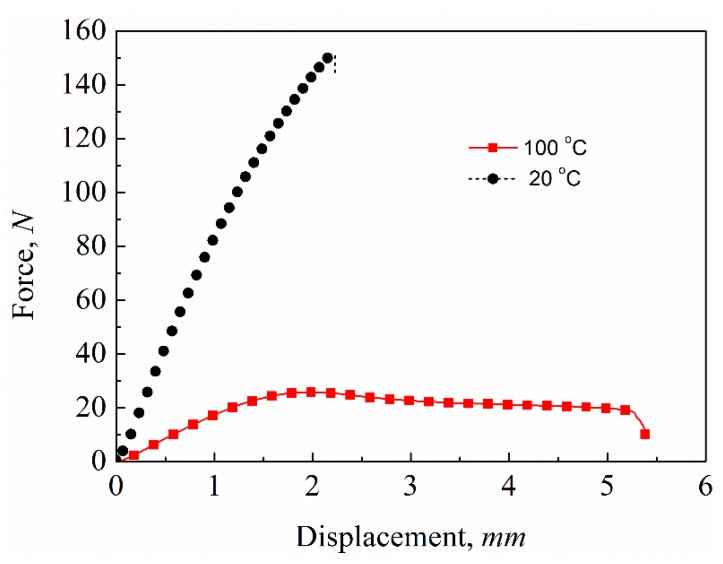
Three-point bending force–displacement curves of variable-stiffness corrugated sheets.

The force–displacement curves of the longitudinal bending test of the variable-stiffness corrugated sheets at different temperatures are shown in Figure 6. As with the transverse tensile stiffness of the corrugated sheet, the longitudinal bending stiffnesses also had good repeatability under the same test conditions. The longitudinal bending stiffness at room temperature, 20 °C, was higher, while the deformation capacity was smaller than that at *T_g_*, 100 °C. The DMA results confirmed the stiffness variation of the SMPE and CFF/SMPE composite. Their storage modulus decreased as the temperature increased and dropped sharply in the *T_g_* region. Thus, the longitudinal bending stiffness of corrugated sheet based on the CFF/SMPE composite at *T_g_*, 100 °C, significantly decreased compared to that at room temperature, 20 °C. The force–displacement curves of the bending test were linear when the mid-span displacement of the corrugated sheet was less than 1.5 mm at both temperatures. The forces dropped and curves became non-linear when the displacement was beyond 1.5 mm. This is because local bucking and damage occurred at the contact point between the corrugated sheet and the test indenter with increased displacement. The aerodynamic pressure load is always treated as a distributed load rather than a concentrated load when the corrugated sheet is used as morphing skin, which can avoid local damage and improve carrying capacity. The mechanical data extracted from three-point bending curves are tabulated in Table 2. The longitudinal bending stiffness of corrugated sheets could be obtained from the linear range, and it was 0.412 ± 0.016 N∙m^2^ at room temperature and 0.076 ± 0.001 N∙m^2^ at *T_g_*. The longitudinal bending stiffness varied 5.4-fold between these two temperatures, which shows the good variable stiffness property of the SMPE composite corrugated sheet. Due to the variable stiffness of the SMPE, the corrugated sheet exhibited a variable stiffness with a change in temperature, which can help the morphing skin adjust its stiffness according to different service environments.

In addition to the variable-stiffness property, the corrugated sheet in this work also displayed extreme mechanical anisotropy resulting from the corrugation. It was flexible in the transverse direction and stiff in the longitudinal direction. The anisotropic mechanical property can be evaluated by the relative transverse stiffness and section bending stiffness [51]. The relative transverse stiffness and section bending stiffness of the corrugated sheet presented in this work could reach 0.0018 and 558.28, respectively, which shows good mechanical anisotropy.

### 3.3. Electrical Heating Performance

**Figure 7:**
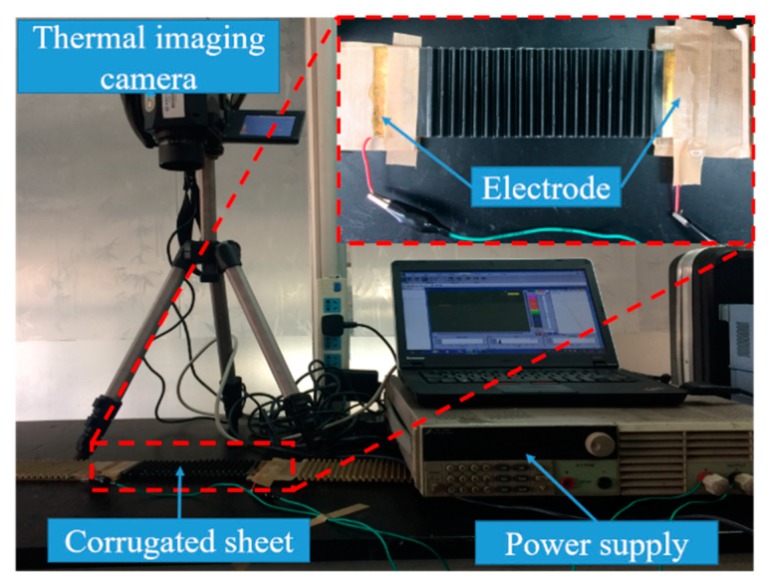
Electrical heating performance test set-up of variable-stiffness corrugated sheet.

**Figure 8:**
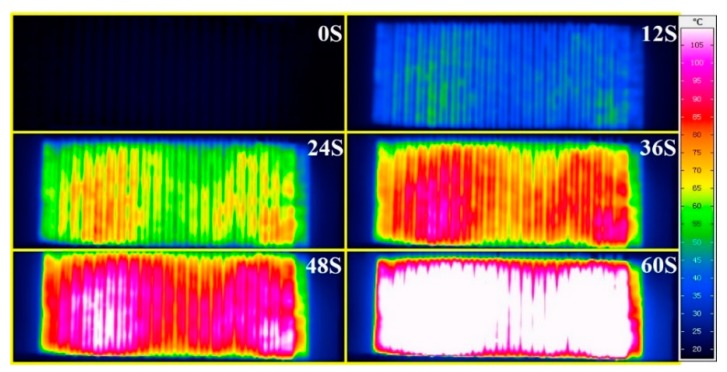
Snapshots of the surface temperature distributions of variable-stiffness corrugated sheet.

The resistance of the CFF/SMPE composite was significantly reduced due to the good conductivity of carbon fiber. Thus, the corrugated sheet based on SMPE composite can be easily triggered by its resistance Joule heat under a small voltage, and its stiffness can be adjusted with the input electric heating power. The electrical heating performance of the corrugated sheet was investigated. The specimen had copper foil electrodes at both ends, and its resistance was 23.1 Ω at room temperature. The dimensions were 160 mm in length, containing 20 corrugation cycles, 3-mm amplitude H/2, and 60-mm width. An infrared camera (VarioCAM^®^ HiRessl, JENOPTIK Infra Tec.) was used to study the temperature distribution on the surface of the corrugated sheet. The layout of the test equipment is shown in Figure 7. The corrugated sheet was pasted 20 mm above the desktop platform to prevent heat conduction between the specimen and the desktop from affecting the test results. An infrared camera was fixed to a tripod to record the temperature distribution of the sheet surface. The surface temperature distribution under a 30-V DC voltage is shown in Figure 8. The surface temperature increases with increased heating time and the surface temperature distribution are uniform. The surface temperature reached 100 °C (*T_g_* of SMPE) at 60 s, which proves that the corrugated sheet can be heated with its resistance Joule heat without an extra heating drive. Considering the stiffness variation with temperature, the corrugated sheet possesses variable stiffness triggered by its Joule heat effect.

**Figure 9:**
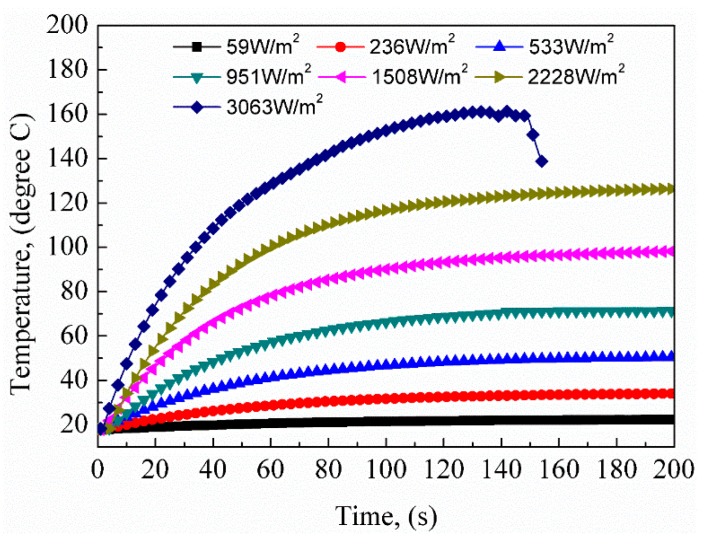
Curves of the average surface temperature versus heating time at different heat flux densities.

The average surface temperature under different voltages was also recorded to investigate the electrical heating performance. The input DC voltages were set between 5 and 35 V with a 5-V interval, and the corresponding heat flux density for each voltage was 59, 236, 533, 951, 1508, 2228, and 3063 W/m^2^. The variation in average surface temperature of the corrugated sheet with heating time is shown in Figure 9. The average surface temperature increased with heating time and maintained an equilibrium temperature after a few seconds when the corrugated sheet was at a thermal equilibrium state. The experimental data for heating performance under different voltages are listed in Table 3. The equilibrium temperature increased with increased heat flux density and the time consumed increased accordingly. The equilibrium temperature was 23 ± 1.6, 32 ± 3.7, 49 ± 2.1, 67 ± 2.5, 92 ± 5.7, and 123 ± 6.7 °C under different heat flux density inputs. The corresponding heating times were 100 ± 8.4, 105 ± 5.4, 116 ± 8.0 s, 123 ± 8.2 s, 132 ± 7.3, and 143 ± 13.1 s. It is worth noting that, when the heat flux density reached 3063 W/m^2^, the average temperature rose rapidly at the beginning and fluctuated after 132 s. The corrugated sheet was thermally ablated and accompanied by smoke at 149 s. To prevent the corrugated sheet from being completely burnt out, the power was immediately cut off; thus, the temperature dropped quickly. This overheating was mainly caused by two reasons—the temperature sensitivity of the SMPE composite’s resistance and the lower thermal conductivity of polymer materials. The CFF-reinforced SMPE composite has a negative temperature coefficient of resistance (NTCR) [28]. Its resistance decreases with increasing temperature. When the temperature of the corrugated sheet rises and remains for a few seconds, its resistance decreases under the influence of high temperature. Due to the decrease in resistance, the heat flux density increases under constant voltage input. The increased heat flux density results in a higher temperature. The reciprocation causes a high temperature, exceeding the material’s temperature capability [25]. With the rapidly rising temperature of carbon fiber, the heat cannot promptly transmit to the polymer due to the low thermal conductivity [26]. The instant high temperature causes the thermal decomposition of the polymer. Therefore, when the corrugated sheet is used as morphing skin, the ambient conditions, including thermal conduction and thermal convection, should be considered to avoid applying excessively high heat flux.

### 3.4. Shape-Memory Properties

**Figure 10:**
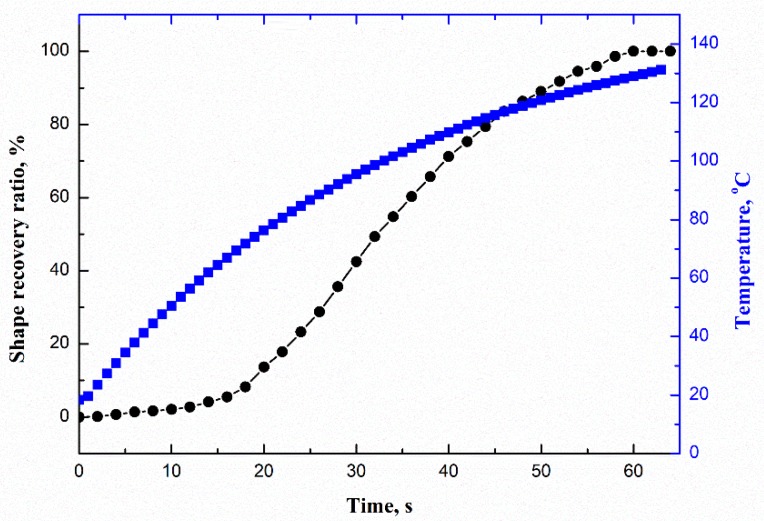
The shape recovery ratio and average temperature over time.

**Figure 11:**
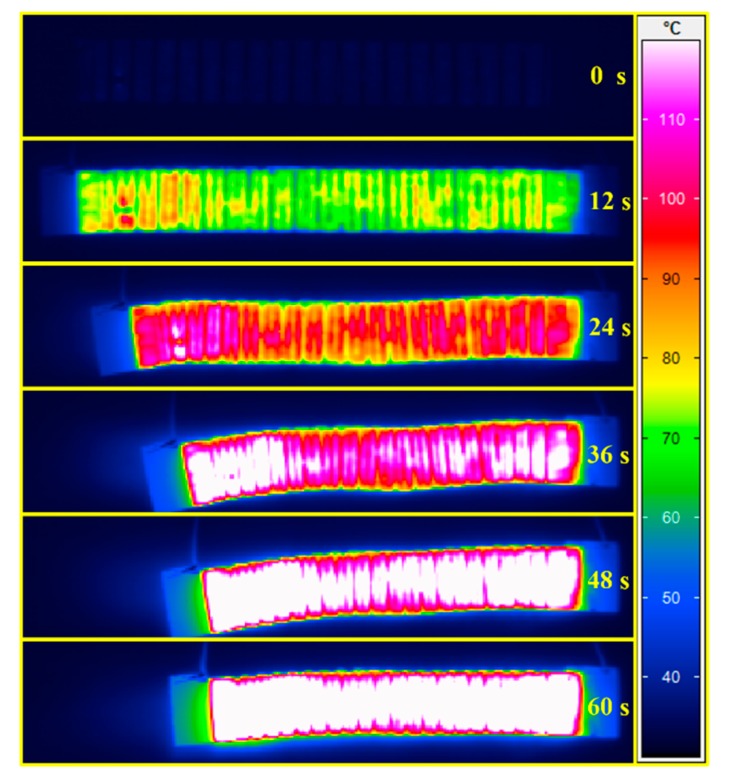
Snapshots of shape recovery and temperature distributions of CFF/SMPE corrugated sheet.

To verify the shape-memory effect (SME) of the corrugated sheet, the tensile shape recovery test using rectangular strip specimens was examined. The specimen had an initial length of L_0_ = 160 mm, which was firstly stretched to L_1_ = 208 mm at 100 °C (*T_g_*), and the stretched temporary shape was locked by cooling it to room temperature. A constant 35-V DC voltage was applied to the two electrodes of the stretched specimen. Meanwhile, the temperature distribution was measured using the infrared temperature camera. The shape recovery ratio and the average temperature of the CFF/SMPE corrugated sheet over time are shown in Figure 10. The entire shape recovery process took 60 s. The average temperature gradually increased over time. In the beginning, the sample needed some time to reach the transition temperature. Thus, the recovery speed was very slow in the first 12 s. After that, the sheet started to recover faster. Starting at 60 s, no visible displacement was noted. The shape recovery process was completed, and the shape recovery ratio was almost 100%, which reveals the good SME of the CFF/SMPE corrugated sheet. The temperature distribution, along with the shape recovery process, is shown in Figure 11. The temperature of the corrugated sheet increased with time. The sample started to shrink rapidly starting at 12 s and completed shape recovery at 60 s. The resistance Joule heat of the CFF/SMPE corrugated sheet can easily trigger its SME without an external heating facility.

## 4. Conclusions

In this work, a variable-stiffness corrugated sheet made of CFF/SMPE composite was fabricated and evaluated with experiments. The investigation focused on mechanical properties, electrical heating performance, and the shape-memory effect. The corrugated sheet shows both excellent variable stiffness properties and extreme mechanical anisotropy, making it a potential choice for morphing skin application. The corrugated sheet can adjust its stiffness according to different service environments that benefit from the variable-stiffness properties. It can be electrically heated rapidly and homogeneously due to its high electrical conductivity and enhanced heat transfer efficiency. The resistance Joule heat can be an effective stimulation of variable stiffness and shape-memory effects. Due to the mechanical anisotropy of corrugation, the corrugated sheets show a low in-plane stiffness to minimize the actuation energy and perform considerable deformation, while they also possess high out-of-plane stiffness to transfer the aerodynamic pressure load.

## Figures and Tables

**Table 1:** The average experimental data of tensile stiffness, force at break, and displacement at break at different temperatures.

Temperature (°C)	Tensile Stiffness (N/mm)	Force at Break (N)	Displacement at Break (mm)
20 °C	1.04 ± 0.06	41.59 ± 4.99	36.36 ± 2.27
100 °C	0.15 ± 0.03	36.74 ± 1.37	108.39 ± 1.19

**Table 2:** The average experimental data of bending stiffness, force at break, and displacement at break at different temperatures.

Temperature (°C)	Bending Stiffness (N∙m^2^)	Force at Break (N)	Displacement at Break (mm)
20 °C	0.412 ± 0.016	151.76 ± 3.34	2.28 ± 0.06
100 °C	0.076 ± 0.001	18.00 ± 1.90	6.15 ± 1.58

**Table 3:** The experimental data of heating performance under different voltages. DC—direct current.

DC Voltage (V)	Heat Flux Density (W/m^2^)	Equilibrium Temperature (°C)	Heat Time (s)
5	59	23 ± 1.6	100 ± 8.4
10	236	32 ± 3.7	105 ± 5.4
15	533	49 ± 2.1	116 ± 8.0
20	951	67 ± 2.5	123 ± 8.2
25	1508	92 ± 5.7	132 ± 7.3
30	2228	123 ± 6.7	143 ± 13.1
35	3063	-	-
